# Sodium-Alginate-Doped Lignin Nanoparticles for PBAT Composite Films to Dually Enhance Tensile Strength and Elongation Performance with Functionality

**DOI:** 10.3390/polym16162312

**Published:** 2024-08-15

**Authors:** Qiyue Guo, Yuan He, Jianyu Wu, Haichuan Ye, Tingting You, Feng Xu

**Affiliations:** 1 Beijing Key Laboratory of Lignocellulosic Chemistry, Beijing Forestry University, Beijing 100083, China; 17864786673@163.com (Q.G.); hy941813@163.com (Y.H.); wjy230485@bjfu.edu.cn (J.W.); haichuan.ye@foxmail.com (H.Y.); xfx315@bjfu.edu.cn (F.X.); 2 Engineering Research Center of Forestry Biomass Materials and Energy, Ministry of Education, Beijing Forestry University, Beijing 100083, China; 3 State Key Laboratory of Biobased Material and Green Papermaking, Qilu University of Technology, Jinan 250353, China

**Keywords:** lignin nanoparticles, PBAT film, elongation at break, UV shielding, moisture barrier

## Abstract

It is a formidable challenge in thermoplastic/lignin composites to simultaneously boost tensile strength and elongation performance due to the rigidity of lignin. To address this issue, sodium-alginate-doped lignin nanoparticles (SLNPs) were prepared by combining solvent exchange and a coprecipitation method and used as an eco-friendly filler for poly(butylene adipate-co-terephthalate) (PBAT). The results indicated that the 1% polyanionic sodium alginate solution contributed to the formation of SLNP in lignin/THF solution. SLNP with a mean hydrodynamic diameter of ~500 nm and a Zeta potential value of −19.2 mV was obtained, indicating more hydrophobic lignin nanoparticles and a smaller number of agglomerates in SLNP suspension. Only 0.5 wt% SLNP addition improved the yield strength, tensile strength, and elongation at break by 32.4%, 31.8%, and 35.1% of the PBAT/SLNP composite films, respectively. The reinforcing effect resulted from the rigid aromatic structure of SLNP, whereas the enhanced elongation was attributed to the nanostructural feature of SLNP, which may promote boundary cracking. Additionally, the PBAT/SLNP composite films displayed excellent ultraviolet (UV) resistance with a UV shielding percentage near 100% for UVB and more than 75% for UVA, respectively. The addition of SLNP hindered water vapor, enhancing the moisture barrier properties. Overall, this study provides an effective strategy to eliminate the decrement in elongation performance for PBAT/lignin composites and suggest they are good candidates to be extensively utilized.

## 1. Introduction

Biodegradable plastics emerge as promising substitutes for conventional, nonbiodegradable plastics to reduce the carbon footprint of these products [1]. It is reported that the global production capacity of biodegradable plastics is predicted to approximately 2.5 million tons per year [2]. Among various biodegradable plastics, poly(butylene adipate-co-terephthalate) (PBAT) is a biodegradable thermoplastic, commonly used as a petroleum plastic replacement in agriculture and packaging fields [3]. PBAT is an aliphatic aromatic co-polyester and has flexile performance with superior processability. While PBAT endows many advantages to plastics, it provides limited UV-blocking and tensile strength properties, making it photodegradable under light irradiation [4,5].

Lignin is one of the most abundant renewable rigid aromatic raw materials in the world, providing rigidity to plant cell walls. Lignin is primarily composed of three phenylpropane lignin units linked via aryl–ether and carbon–carbon bonds and has ideal properties for lignin valorization, such as ultraviolet (UV) blocking, anti-bacterial, and anti-oxidant properties [6,7,8]. The use of lignin in PBAT plastics is therefore a feasible and cost-effective way of using PBAT’s multi-functional properties, improving durability in application in the packaging and mulching fields [9,10,11,12]. For instance, Xiong et al. blended PBAT with methylated technical lignin and the prepared maleic anhydride graft PBAT compatilizer to prepare economically feasible and strong PBAT/lignin composites [9]. As reported by Kim et al. [10], the oxygen and moisture barrier as well as the UV properties of PBAT films were all enhanced when adding acetylated lignin into the PBAT films. Despite the enhancement in UV-blocking properties, poor compatibility with PBAT often reduces the elongation performance of all PBAT/lignin composites in addition to other mechanical properties for composites with high lignin loading. Recently, lignin at the nanoscale, known as lignin nanoparticles (LNPs) or colloidal lignin particles, has shown great potential in overcoming the phase separation obstacles in lignin/PBAT composites. The tensile performance of LNP-filled PBAT composites improved with low filler loading. Nevertheless, the elongation at break still either decreased or increased slightly [11,12]. In contrast, in an early report, it was shown that 5 wt% hydrophobic lignin reverse micelles, prepared from an alkali lignin/dioxane solution using 7 vol % cyclohexane as an exchange solvent, improved the mechanical performance of high-density polyethylene as well as the elongation at break [13]. Thus, hydrophobic LNPs prepared using different methods may have a significant influence on the mechanical properties of thermoplastic/LNP composites. Therefore, developing a simple, safe, and applicable fabrication strategy for hydrophobic LNP to meet the needs of dually improved tensile strength and elongation performance is urgent.

Existing LNP preparation methods have been broadly developed and thoroughly reviewed [14,15]. In general, these methods can be categorized into five groups: antisolvent precipitation, solvent exchange, sonication, mechanical treatment, and others. Among these methods, solvent exchange is promising and is considered to be environmentally friendly due to the use of a limited amount of organic solvents, forming LNPs at a neutral pH [14,16,17,18]. The initial lignin concentration and water addition rate determine the water content in the mixture, leading to the formation of different sizes of LNPs. Wang et al. demonstrated that, as the water content increased, the hydrophilicity in lignin molecules increased [18]. At the molecular scale, it is reported that improving the homogeneity of electrostatic forces in lignin molecules contributes to the formation of monodispersed lignin colloidal spheres [18]. Enlarging the electrostatic interaction is therefore encouraged in improving the hydrophobicity of LNPs.

**Figure 1:**
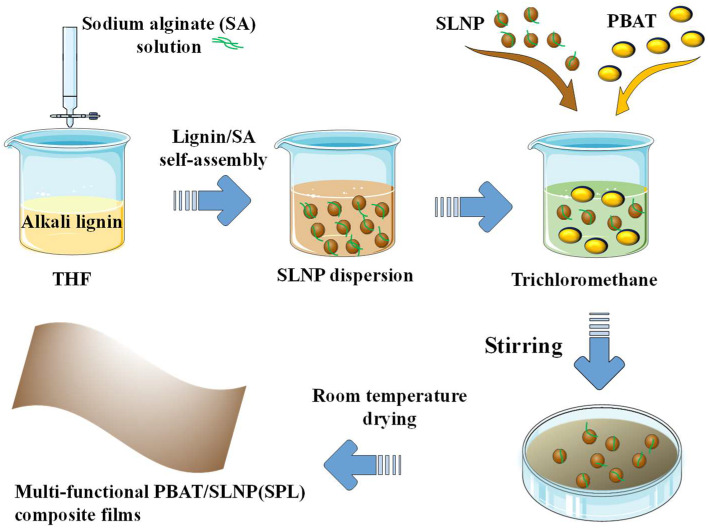
Flowchart of SLNP and multi-functional PBAT/SLNP composite (SPL) film preparation.

In this work, 1 wt% sodium alginate (SA) solution is first added into an alkali lignin/tetrahydrofuran (THF) solution by solvent exchange, leading to self-assembly of sodium alginate to form sodium-alginate-doped LNPs (SLNPs), used as an eco-friendly filler for PBAT (Figure 1). Sodium alginate is a polyanionic polysaccharide in nature [19]. On the one hand, the polyanionic character may enhance the homogeneity of electrostatic forces to promote SLNP formation. On the other hand, sodium alginate will coagulate in THF and coprecipitate with LNPs. The characteristics of SLNP, as well as the properties of PBAT/SLNP composite (SPL) films, were comprehensively investigated and compared with those of water-regenerated LNPs (WLNPs) and the corresponding composite (WPL) films. This study provides an innovative design strategy for the simultaneous strengthening and toughening of PBAT/lignin composite films with functionality.

## 2. Materials and Methods

### 2.1. Materials

Alkali lignin was obtained from Shandong Longlive Co., Ltd., Dezhou, China. PBAT (MW 30,000 Da) was purchased from Xinjiang Blue Ridge Tunhe Sci. & Tech. Co., Ltd., Changji, China. Sodium hydroxide (CAS No. 1310-73-2, AR, 96%), hydrochloric acid (CAS No. 7647-01-0, AR, 37%), trichloromethane (CAS No. 67-66-3, contains ethanol as stabilizer, ACS reagent, ≥99.8%), tetrahydrofuran (CAS NO.109-99-9, AR, 99.0% with 250 ppm BHT stabilizer), and sodium alginate (CAS No.9005-38-3, AR, 90%) were acquired from Sinopharm Chemical Reagent Co., Ltd., Beijing, China, and these were used without further purification.

### 2.2. Preparation of Purified Lignin

A measure of 20 g of alkali lignin was added to a beaker containing 380 mL of 1 mol/L NaOH solution and stirred at room temperature to uniformly dissolve the lignin. After dissolution, the prepared solution was centrifuged at a rate of 10,000 r/min for 20 min to remove insoluble impurities. A measure of 1 mol/L HCl solution was then added drop by drop to the supernatant until a solution with a pH of 2 was reached, allowing the lignin to precipitate. The lignin precipitation was washed thoroughly to reach a neutral state, and it was vacuum-dried at 45 °C to obtain the purified lignin.

### 2.3. Preparation of Lignin Nanoparticles (LNPs)

A measure of 100 mg of lignin was dissolved in 10 mL of tetrahydrofuran and stirred at 500 rpm at room temperature for 1 h. Measures of 80 mL of the following antisolvents were added by peristaltic pump at a rate of 10 mL/h: deionized water; 1 wt% sodium alginate solution. Then, the water content in the mixture was adjusted by the total dropping time (2 h, 4 h, 6 h, and 8 h). THFs were removed by stirring overnight, and then the mixture was centrifuged at 10,000 r/min for 30 min. Then, the supernatant was removed, and the resulting precipitate was washed with water until it reached a neutral state. The precipitate was freeze-dried overnight to obtain water-regenerated LNPs (WLNPs) and SA-regenerated LNPs (SLNPs).

### 2.4. Preparation of PBAT/LNP Composite Films

PBAT/LNP composite films were prepared using a solution-casting method. The procedure was as follows: PBAT (5.00 g) was dissolved in 60 mL of trichloromethane, and 0, 0.5 wt% (25 mg), 1 wt% (50 mg), and 1.5 wt% (75 mg) of LNPs were dispersed in 20 mL measures of trichloromethane, respectively. The LNPs dispersion was added slowly to the PBAT solution under mechanical stirring for 30 min, followed by ultrasonication to homogeneously disperse the lignin. The resulting mixture was then poured into a glass Petri dish. The composite films were obtained after evaporation of the solvent at room temperature. The thickness of the composite films was 80 ± 6 μm. These films were referred to as WPLx and SPLx, where x is the amounts of WLNPs and SLNPs in the composite films, respectively.

### 2.5. Characterizations

#### 2.5.1. Morphology

The morphology of the LNPs, the cross-sections, and the surfaces of the PBAT/LNP composite films was observed using field emission–scanning electron microscopy (FE-SEM) (SU8010, Shimadzu Co., Kyoto, Japan) at acceleration voltages of 3.0 kV.

#### 2.5.2. Zeta Potential and Particle Size Analysis

The zeta potential and corresponding particle size of LNPs were measured using an ALV/DLS/SLS-5022 F instrument (ALV/Laser Vertriebsgesellschaft m.b.H Co., Blomberg, Germany) in ultrapure water at pH 7.0 and with a concentration of about 0.10 mg/mL.

#### 2.5.3. Contact Angle Measurement

A contact angle analyzer (SL200KS, Kino Co., Boston, MA, USA) was used for the contact angle measurement of the LNPs powder. The powder sample was uniformly attached to a double-sided adhesive tape fixed on a slide, followed by gently tapping the slide to remove excess powder adhering to and around the sample surface. Each sample was measured in triplicate.

#### 2.5.4. Properties of PBAT/LNP Composite Films

##### Functional Groups

The functional groups in the PBAT/LNP composite films were analyzed by Fourier transform infrared (FT-IR) spectroscopy (Tensor 27, Bruker Co., Saarbrucken, Germany) in the transmission mode in a range of 4000–400 cm^−1^. KBr pellets containing 1% ground samples were used.

##### Mechanical Properties

The dimensions of the sliced films were 15 mm width and 80 mm length; the slice films were subjected to mechanical testing using a Zwick/Roell apparatus (Z005, Zwick Co., Schweinfurt, Germany) equipped with a 200 N capacity load cell. The crosshead was operated at a rate of 25 mm/min under room temperature. Prior to testing, the film was allowed to stand at a constant relative humidity of 53% for 24 h and the distance between the clamps should be maintained at 50 mm before testing commenced. The tensile test was then carried out at a speed of 300 mm/min.

##### Moisture Barrier Properties

The PBAT/LNP composite films were evaluated using a specialized tester (Model W3/031, Labthink Instruments, Jinan, China) adhering to a modified version of the ASTM E96/E96M-16 standard [20], to assess their moisture barrier performance. Each sample’s water vapor transmission rate (WVTR) values were calculated using Formula (1) and measured three times for accuracy and precision.

(1) $$WVTR=\frac{Sample\;weight\;changes}{Area\;\times \;time}$$

##### UV Resistance

The UV–visible spectrophotometer, augmented with an integrating sphere attachment (UV-2550, Shimadzu, Kyoto, Japan), was utilized to assess the UV shielding capabilities of the films. Specifically, different film samples measuring 4 cm × 3 cm were tested and then Equations (1) and (2) [21] were used to quantify the resistance to UVB (280–320 nm) and UVA (320–400 nm), respectively:

(2) $$\mathrm{UVB}\;\mathrm{blocking}\;(\%)=100\;-\frac{\int_{\;280}^{320}T(\lambda )d(\lambda )}{\int_{\;280}^{320}d(\lambda )}\times 100$$

(3) $$\mathrm{UVA}\;\mathrm{blocking}\;(\%)=100\;-\frac{\int_{\;320}^{400}T(\lambda )d(\lambda )}{\int_{\;320}^{400}d(\lambda )}\times 100$$

The term T(λ) signifies the mean spectral transmittance across the film samples, whereas d(λ) corresponds to the spectral width or bandwidth, and λ designates the specific wavelength being examined. This notation facilitates the calculation of optical properties in a precise and scientific manner.

##### Thermal Stability

The thermogravimetric analysis (TGA) was performed on TGA/SDTA851e apparatus from Mettler–Toledo. Approximately 10 mg in weight of each sample was used. The heating process was as follows: initiated temperature (25 °C); heating rate (10 °C per minute); all under a continuous flow of nitrogen gas.

## 3. Results and Discussion

### 3.1. Characterization of SLNPs

**Figure 2:**
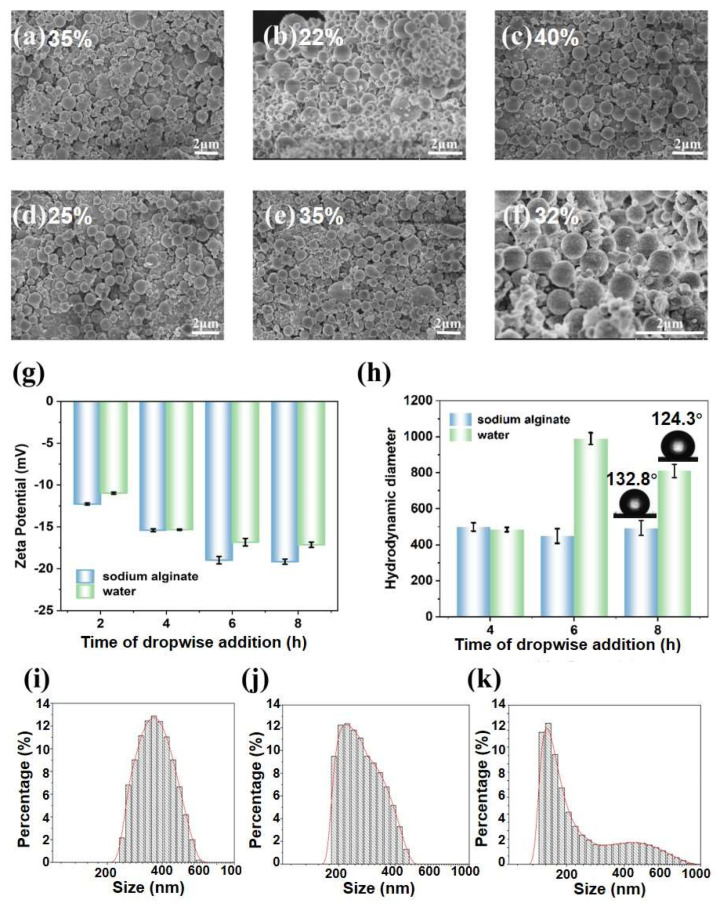
SEM images and the yields of LNPs prepared by dropwise addition of different concentrations of sodium alginate and water: (**a**) 0.25%, (**b**) 0.5%, (**c**) 1%, (**d**) 1.5%, (**e**) 2%, and (**f**) water. (**g**) Zeta potential of SLNP and WLNP prepared at different dropwise addition durations. (**h**) The mean hydrodynamic diameter of LNPs under different dropping times. Particle size distribution of SLNPs for time of dropwise addition for 4 h (**i**), 6 h (**j**), and 8 h (**k**).

The SLNPs were prepared using a solvent exchange and coprecipitation mechanism. The effects of sodium alginate (SA) concentration on nanoparticle formation were investigated. The SEM images of SLNP prepared from 0.25 wt% (Figure 2a), 0.5 wt% (Figure 2b), 1 wt% (Figure 2c), 1.5 wt% (Figure 2d), and 2 wt% (Figure 2e) sodium alginate solution and deionized water addition (Figure 2f) are shown. As can be seen, the concentration of sodium alginate solution affected the surface properties. Specifically, using low concentrations (e.g., 0.25% and 0.5%) of sodium alginate may not be sufficient to form LNPs, resulting in less effective dispersion and stabilization of the particles, and many tiny fragments were found. In contrast, more blocky structures were observed when adding a high concentration of SA (e.g., 1.5% and 2%) due to the coagulation of SA to LNPs [19]. Optimally, SLNP prepared using 1% concentration of sodium alginate showed a narrow size distribution with highest yield (40%). This suggested that 1% SA can be moderately adsorbed on the surface of the lignin nanoparticles to form an effective protective layer, which not only prevents direct contact and agglomeration between the particles, but also maintained a good mobility, allowing the lignin nanoparticles to be uniformly dispersed and stabilized during the preparation process [22,23]. SEM-EDS mapping results of Na element further confirmed the successful introduction of sodium alginate (Figure S1). An SA concentration of 1% with highest yield (40%) was therefore selected for further research.

The SLNPs and WLNPs obtained under the same dropwise addition interval duration were investigated to reveal the effects of water content in the mixture solutions on LNPs formation. The Zeta potential value of LNPs increased with the time of the dropwise addition, and the SLNPs showed a higher Zeta potential value than the WLNPs under each dropwise addition duration (Figure 2g). This phenomenon indicated that the addition of sodium alginate may strengthen the electrostatic repulsion of the negatively charged groups in the sodium alginate molecules with the lignin molecules, resulting in an increase in surface charge. The highest Zeta potential value of −19.2 mV for SLNP-8 h was obtained, which was higher than that of the LZ nanoparticles [12]. The mean hydrodynamic diameter of the SLNPs was about 500 nm, which is smaller than that of WLNP (Figure 2h), indicating that a smaller number of agglomerates were dispersed in the SLNP liquid. The hydrodynamic diameter distribution analysis demonstrated wider size distribution but smaller sizes were found for SLNP-8 h (Figure 2k) than others (Figure 2i,j). The average polydispersity indice (PDI) values were 0.24, 0.30, and 0.22 for SLNP-4 h, SLNP-6 h, and SLNP-8 h, respectively. The decrease in particle size may be ascribed to the present of more hydrophobic lignin in SLNP-8 h. SLNP-8 h was therefore chosen for further study. The water contact angle of SLNP-8 h was 132.8°, which was higher than that of the WLNPs (Figure 2h). The higher hydrophobicity of the SLNPs may contribute to a higher mechanical property for thermoplastic films.

### 3.2. Characterization of PBAT/LNP Composite Films

**Figure 3:**
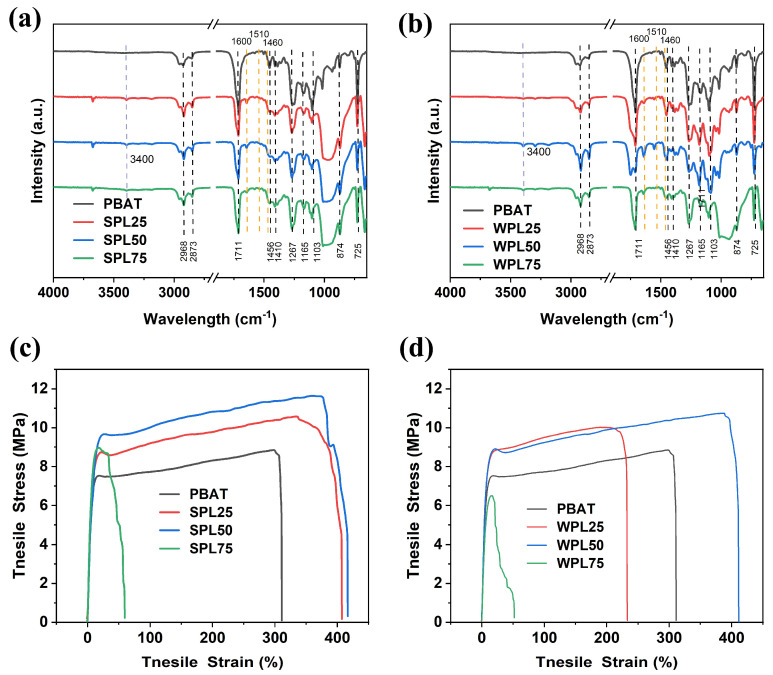
The FT-IR spectra of (**a**) SPL films and (**b**) WPL films. Stress–strain curves of the (**c**) SPL films and (**d**) WPL films.

The FT-IR spectra of the pure-PBAT films, the PBAT/SLNP composite (SPL) films, and the PBAT, PBAT/WLNP composite (WPL) films are shown in Figure 3a,b, respectively. All of the films show characteristic peaks of PBAT, located at 2968, 2873, 1711, 1456, 1410, 1267, 1165, 1103, 874, and 725 cm^−1^. Among them, the peaks at 1711 cm^−1^ correspond to the C=O double bonds, whereas the absorption peaks in the range of 1103–1267 cm^−1^ are due to ether linkages (C–O–C) in PBAT [24]. In the spectra of SPL, new peaks at 3400 cm^−1^ appeared, which correspond to the vibration of the OH group. It is known that alginate is a natural polysaccharide consisting of D-mannuronate and L-guluronate. The new peaks may result from the OH group in SA [25], confirming the successful doping of SA in SLNP. Despite the fact that the LNPs were dispersed in the films, the absorption peaks for lignin at around 1460 cm^−1^, 1510 cm^−1^, and 1600 cm^−1^ were too weak to observe; these were assigned to the aromatic backbone vibrations in lignin [26].

The mechanical properties of the pure-PBAT and PBAT/LNP composite film materials were tested, and the results are shown in Figure 3c,d and Table 1. As can be seen from Figure 3c,d, the whole stress–strain process of SPL and WPL can be divided into three stages: yielding, orientation, and fracture. The pure-PBAT film exhibits good mechanical properties, with a yield strength of about 7.4 MPa and a tensile strength of 8.8 ± 0.6 MPa (Table 1). Both the yield strength and the tensile strength of the composite film materials obtained by adding LNPs firstly increased and then decreased. Especially noteworthy is the fact that the yield strengths of SPL25 and SPL50 reached 9.0 and 9.8 MPa, with an increment of 21.6% and 32.4%, respectively. As listed in Table 1, with only 0.25 and 0.50 wt% addition of SLNPs, the tensile strength increased from 8.8 ± 0.6 MPa for the pure-PBAT film to 10.6 ± 0.6 and 11.6 ± 0.7 MPa, respectively. The increases in yield and tensile strength was due to the rigid aromatic structure of lignin. However, when the SLNP loading content increased to 0.75 wt%, the tensile strength decreased clearly. This phenomenon may be attributed to the aggregation of SLNPs, which makes SLNPs hard to disperse well in the PBAT/LNPs composite films. A similar tendency was found for the elongation at the break. The SLNPs showed toughening effects when the SLNP loading contents were 0.25 and 0.50 wt%. The nanostructural feature of the SLNPs may promote boundary cracking by producing a large bending case at the crack front end. However, a drastic reduction in the elongation at break was found for SPL75. This was also attributed to the aggregation of SLNP, giving a poor compatibility. It is noted that the composite films with SLNP addition exhibited improved tensile strength and elongation at the break than those with WLNP addition. It is well known SA is a kind of polysaccharide which is rich in the hydroxyl group. More flexible hydrogen bonds between lignin and SA may be formed to make SLNPs more elastic and strong than the WLNPs, enhancing the mechanical strength of the composite films. It is inferred that the tensile strength and elongation at the break of SPL50 increased by 31.8% and 35.1%, respectively. The enhancement was higher than that of WPL and the reported ones [9,10,12,27] (Table 2). The maintaining of the elongation performance of PBAT when adding lignin is attractive for PBAT in the mulching and packaging fields.

**Figure 4:**
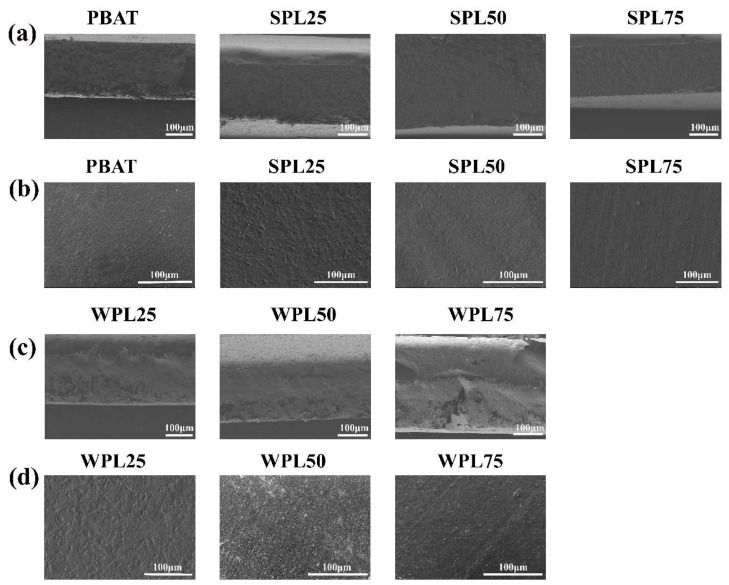
SEM of cross-section of (**a**) SPL films and (**c**) WPL films and the surface of (**b**) SPL films and (**d**) WPL films.

An SEM analysis was carried out to observe the cross-sections and surfaces of the composite films (Figure 4). Specifically, the SLP50 and SLP75 films exhibited a smoother and more compact cross-sectional structure than those of the WLP films, in which more voids and a looser structure were observed. This difference was attributed to the enhancement of the interaction among the lignin nanoparticles by the introduction of sodium alginate. The molecular chain of sodium alginate is rich in OH and COO^−^ groups [25], which can effectively promote hydrogen bonding and electrostatic interaction between the lignin nanoparticles, resulting in a tighter and more organized structure during the film-formation process. In addition, sodium alginate may improve the fluidity during the film preparation process, allowing the lignin nanoparticles to uniformly distribute in the substrate and reducing the formation of voids. For the films surface, the SLP50 films showed the smoothest morphology, while the other films were relatively rough. This result further confirmed the key role of sodium alginate in optimizing the microstructure of the films, which not only strengthened the internal structure of the films, but also significantly improved the surface quality of the films.

**Figure 5:**
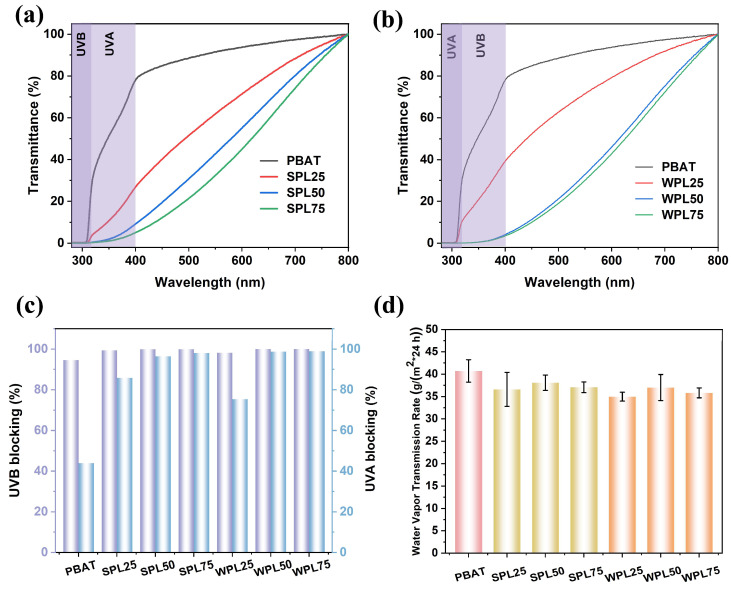
UV–vis spectra showing transmittance of (**a**) SPL films and (**b**) WPL films, (**c**) UVB- and UVA-blocking rate, and (**d**) water vapor permeability of the multifunctional PBAT/LNP composite films.

The aromatic ring ketones, quinones, and phenol structures offer lignin with a UV-absorbing capacity, making it an ideal alternative to commercial UV-blocking materials [27,28,29]. The optical transmittance of PBAT/LNP films at different wavelengths was further investigated to evaluate the UV-blocking property. As shown, the absorbance in the ultraviolet region of the SPL and WPL films decreased continuously when compared to the pure one, showing a good ultraviolet shielding performance (Figure 5a,b). All of the SLP and WLP films can block nearly 100% of UVB (Figure 5c) and over 75% of UVA (Figure 5d), which is much higher than the blocking rate of PBAT. It is noted that the WPL films had slightly higher UVA-blocking rates than the SPL films. This phenomenon may be due to the doping of SA on the surface of the lignin nanoparticles. Owing to the inherent coloration of lignin, there is a decrement in the optical transmittance across the visible light spectrum (ranging from 400 to 800 nm), but a notable level of light permeability is sustained. The transmittance values of SLP and WLP at the characteristic wavelength of 550 nm were 30.1~60.4% and 29.8~70.6%, respectively.

Additionally, the water vapor transmission rate (WVTR) values of the PBAT/LNP composite films decreased when compared to those of PBTA, indicating an enhanced moisture barrier property. These are consistencies between these findings and those of previous reports [11,12]. All of the barrier properties induced by LNPs endow PBAT with functionality. The versatile PBAT/LNP composite films may facilitate the realization of mulching and package films for practical applications.

**Figure 6:**
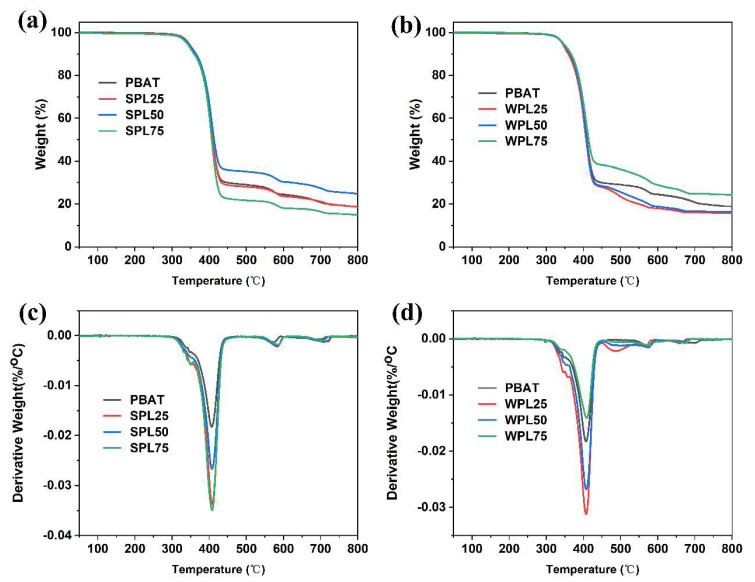
TGA curves of (**a**) SPL films and (**b**) WPL films, and (**c**), (**d**) DTG curves of (**c**) SPL films and (**d**) WPL films.

The thermal degradation properties of SLP and WLP films were analyzed using the thermogravimetric technique, and the TGA and DTG results are shown in Figure 6a–d, respectively. As shown, the incorporation of LNPs can slightly improve the thermal stability properties of the films. This is a result of the higher thermal resistance of lignin induced by the aromatic carbon. The improved thermal stability of the composite films will guarantee their versatile applications in harsh environments. Moreover, both PBAT and lignin are known as typical biodegradable polymers, suggesting the biodegradability of composite films under normal conditions.

## 4. Conclusions

In summary, we investigated the application of sodium-alginate-doped lignin nanoparticles (SLNPs) to enhance the tensile strength and the elongation performance, and endowed the multi-functional properties of the resulting films. SLNP was first prepared by solvent exchange and coprecipitation of alkali lignin from a 1% sodium alginate–THF mixture. It is evidenced from FT-IR and SEM analysis that the SA was successfully incorporated in lignin nanoparticles. Compared to WLNP, SLNP was more hydrophobic and smaller in mean hydrodynamic diameter, contributing to a higher mechanical property for thermoplastic films. The incorporation of SLNP into PBAT resulted in tighter and more organized structures in PBAT/SLNP composite films. Consequently, the yield strength, tensile strength, and elongation at break of 0.5 wt% SLNP adding PBAT composite (SPL50) films increased by 32.4%, 31.8, and 35.1%, respectively. The enhanced yield and tensile strength were attributed to the rigid aromatic structure of SLNP; whereas the improved elongation was ascribed to the nanostructural feature of SLNP, probably promoting boundary cracking. Taking advantage of SLNP, the PBAT/SLNP composite films had UV resistance and moisture barrier properties, while having retained or improved mechanical performances. This work not only paves the way for preparing more hydrophobic lignin nanoparticles but also expands their potential applications in biodegradable thermoplastic films with tailored functionalities.

## Figures and Tables

**Table 1:** Tensile strength and elongation performance of PBAT/LNP composite films in this work.

Samples	Composition	Tensile Strength (MPa)	Elongation at Break (%)
SLP25	PBAT, 0.25% SLNP	10.6 ± 0.6	394 ± 35.4
SLP50	PBAT, 0.5% SLNP	11.6 ± 0.7	404 ± 42.2
SLP75	PBAT, 0.75% SLNP	9.5 ± 0.3	44 ± 28.4
WLP25	PBAT, 0.25% WLNP	10 ± 0.5	220 ± 37.6
WLP50	PBAT, 0.5% WLNP	10.7 ± 0.4	398 ± 39.8
WLP75	PBAT, 0.75% WLNP	6.5 ± 0.3	16 ± 9.2
PBAT	PBAT	8.8 ± 0.6	299 ± 23.8

**Table 2:** Comparison of the tensile strength and elongation performance of PBAT/lignin composite films.

Samples Composition	TS	TS Increment	Elongation at Break	Elongation at Break Increment	References
PBAT, 2% LZM	Increased from 33.2 to 37.6 MPa.	+13%	Decreased from 1100 to ~1048%	−4.73%	[12]
PBAT, 0.5% LNP	Increased from 22.6 ± 1.2 to 24.6 ± 0.6 MPa	+9%	Increased from 1450 ± 80 to 1520 ± 20%	+4.82%	[27]
PBAT, 30% acylated lignin	Decreased from 29.8 ± 2.2 to 20.9 ± 1.5 MPa	−29.9%	Decreased from 879 ± 48 to 692 ± 67%	−21.3%	[10]
PBAT, 3% MP, and 60% lignin	Decreased from 23.7 ± 1.5 to 16.7 ± 0.55 MPa	−29.5%	Decreased sharply from 816.49 ± 52.95 to 49.26 ± 5.34%	−93.5%	[9]
PBAT, 0.5% SLNP	Increased from 8.8 ± 0.6 to 11.6 ± 0.7 MPa	+31.8%	Increased from 299 ± 23.8% to 404 ± 42.2%	+35.1%	In this work

## Data Availability

The original contributions presented in the study are included in the article/supplementary material, further inquiries can be directed to the corresponding author.

## Supplementary Materials

The following supporting information can be downloaded at: https://www.mdpi.com/article/10.3390/polym16162312/s1, Figure S1: SEM-EDS mapping results of Na element.
