# Supplementary material for: Sodium-Alginate-Doped Lignin Nanoparticles for PBAT Composite Films to Dually Enhance Tensile Strength and Elongation Performance with Functionality
Source: Polymers (Basel). 2024 Aug 15;16(16):2312. doi: 10.3390/polym16162312 (PMC11359584; doi:10.3390/polym16162312)
Supplement: Supplementary file 1 [file polymers-16-02312-s001.zip › polymers-3142570-supplementary.pdf]

Supporting information for

Sodium-Alginate-Doped Lignin Nanoparticles for PBAT Composite Films to Dually Enhance Tensile Strength and Elongation Performance with Functionality

Qiyue Guo [1,2,†], Yuan He [1,2,†], Jianyu Wu [1,2], Haichuan Ye [1,2], Tingting You [1,2,3,*] and Feng Xu [1,2,3]

[1] Beijing Key Laboratory of Lignocellulosic Chemistry, Beijing Forestry University, Beijing 100083, China; 17864786673@163.com (Q.G.); hy941813@163.com (Y.H.); wjy230485@bjfu.edu.cn (J.W.); haichuan.ye@foxmail.com (H.Y.); xfx315@bjfu.edu.cn (F.X.)
[2] Engineering Research Center of Forestry Biomass Materials and Energy, Ministry of Education, Beijing Forestry University, Beijing 100083, China
[3] State Key Laboratory of Biobased Material and Green Papermaking, Qilu University of Technology, Jinan 250353, China
* Correspondence: youtingting0928@bjfu.edu.cn
† These authors contributed equally to this work.

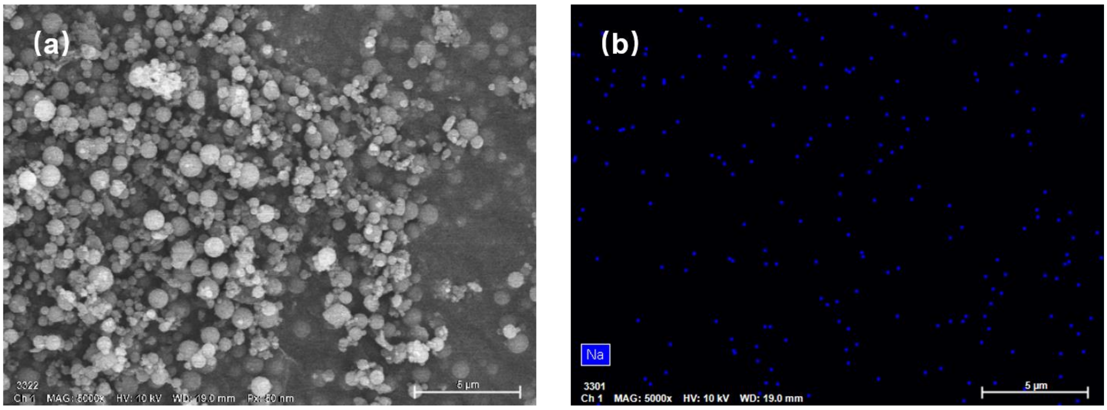

Figure S1. The SEM image (**a**) and SEM-EDS mapping (**b**) of Na element of SLNP.
